# More beds are not the answer: transforming detoxification units into medication induction centers to address the opioid epidemic

**DOI:** 10.1186/s13722-017-0092-y

**Published:** 2017-11-15

**Authors:** Peter D. Friedmann, Joji Suzuki

**Affiliations:** 1 Office of Research and Department of Medicine, University of Massachusetts-Baystate and Baystate Health, Springfield, MA USA; 2 Division of Addiction Psychiatry, Department of Psychiatry, Brigham and Women’s Hospital, Boston, MA USA

Given extensive research evidence that pharmacotherapy is the most effective treatment for opioid use disorder (OUD), and the unabating rise in opioid overdose deaths, it is increasingly apparent that detoxification and “drug-free” treatment should be replaced by medication induction and long-term pharmacotherapy as the first-line standard of care. Nonetheless, calls for more “treatment beds” are heard in the political and public health discourse surrounding interventions to address the epidemic of opioid overdose deaths. States seek federal Medicaid waivers to expand treatment beds, and legislatures increase funding for state-sponsored beds. The term “beds” is usually shorthand for detoxification beds, short-term inpatient or residential beds, and may include outpatient treatment with beds in sober housing, but it usually does not refer to slots in opioid treatment programs or other settings that utilize effective medication treatment for opioid use disorder (OUD). To access these “beds”, patients with OUD first undergo detoxification, whether in an inpatient detoxification program, jail or hospital, and are then discharged to these settings where they receive monitoring, psychosocial support and counseling, but typically no effective medication.

This system of care (i.e. detoxification followed by psychosocial treatment) has traditionally been called “abstinence-oriented” or “drug-free” treatment. However, it is more accurately characterized as “medication-free” treatment [1]. Despite the robust body of research evidence demonstrating improved clinical outcomes with pharmacotherapy, the majority of these programs do not provide addiction medication [2]. These medication-free programs, their patients and communities usually adhere to the recovery-oriented tradition, for which the use of medication is often stigmatized as inconsistent with “true recovery”. As a result, many appropriate patients do not receive effective medications for OUD, and even those who are doing well on medication commonly experience and internalize pressure to “detoxify” off their medication. For example, initiation of the 12-steps, as well as attendance at 12-step meetings, is a common element in most medication-free programs, and 12-step adherents commonly pressure patients with OUD to discontinue medication treatment within artificial time constraints [3]. This situation is unfortunate given that many in the abstinence-oriented treatment community have recognized that medication treatment is not incompatible with recovery [4]. The premature discontinuation of pharmacotherapy is especially troubling given the substantial increase in overdose mortality immediately after cessation of effective opioid agonist treatment [5].

Opioid agonist treatment reduces recurrent illicit opioid use, and maintains tolerance to opioids, which together protect against overdose. Detoxification and medication-free treatment cause patients with OUD to lose their tolerance. In doing so, detoxification and medication-free treatment set up these patients for overdose and death when they return to opioid use [6–8], which they do in the majority of cases—the annual rate of recurrent opioid use is over 60% in the absence of ongoing medication [9–11]. This chain of events (i.e. detoxification, loss of tolerance to opioids, psychosocial treatment with limited effectiveness for OUD, and recurrent opioid use), accounts for high rates of overdose and death after patients with OUD leave incarceration or detoxification [6, 12].

The medical literature comparing detoxification plus psychosocial treatment versus long-term opioid agonist treatment goes back decades, and is remarkably consistent. Clinical outcomes for OUD are best while medication is continued, then the decay is precipitous once the pharmacotherapy is stopped, regardless of whether the taper is short, long or in-between [7, 13–16], and whether or not accompanied by intensive counseling and services [17, 18]. The same likely applies for antagonist treatment—the benefits of extended-release naltrexone (XR-NTX) for OUD dissipated soon after the active treatment period ended in a recent clinical trial [19]. Also, a recent reanalysis of that study found a 30-day relapse rate of only 7% for patients who received XR-NTX upon discharge from a short-term inpatient program, compared to 63% for those released without the medication [20]. The lower recurrence rates among OUD patients receiving medications also reduces costs. In Massachusetts, for example, total health expenditures per month among OUD patients receiving addiction medications was $153–$233 lower than those receiving only psychosocial treatment [21].

Taken together, the available evidence suggests that for opioid use disorders, the risk of recurrent use, overdose and death is high if patients are withdrawn from, or do not receive, effective long-term medication treatment. The standard for what constitutes acceptable first-line care for OUD thus needs to be reconsidered. Tapering off addiction medication may be possible for some patients at some point, but should never be a goal and always subordinate to maintaining safe remission. To address the epidemic of OUD and related deaths, rather than just building more detoxification and medication-free treatment beds, state and federal funding should be predicated on programs and clinicians encouraging OUD patients to initiate and maintain effective long-term medication treatment.

For OUD, “detoxification units” should be reengineered into “medication induction centers” in which patients can select among the increasing number of effective medication options for OUD and have them initiated in a controlled environment [20]. These induction centers would serve as hubs in a hub-and-spoke arrangement that links patients to long-term programs that use effective medication treatment for OUD. Adoption of the hub-and-spoke approach led to a 64% increase in the number of Vermont physicians waivered to prescribe buprenorphine and a 50% increase in OUD patients per waivered physician, giving the Green Mountain state the highest OUD treatment capacity in the U.S. [22]. In transforming the system of care for OUD to conform to evidence rather than tradition, we will learn that what is needed is not more beds, but more outpatient medication treatment slots, at a considerably lower cost and greater effectiveness.
